# Author Correction: G9a regulates breast cancer growth by modulating iron homeostasis through the repression of ferroxidase hephaestin

**DOI:** 10.1038/s41467-020-17413-z

**Published:** 2020-07-24

**Authors:** Ya-fang Wang, Jie Zhang, Yi Su, Yan-yan Shen, Dong-xian Jiang, Ying-yong Hou, Mei-yu Geng, Jian Ding, Yi Chen

**Affiliations:** 1 Division of Anti-Tumor Pharmacology, State Key Laboratory of Drug Research, Shanghai Institute of Materia Medica, Chinese Academy of Sciences, Shanghai, 201203 China; 2 University of Chinese Academy of Sciences, No. 19A Yuquan Road, Beijing, 100049 China; 3 Key Laboratory of Systems Biology, CAS Center for Excellence in Molecular Cell Science, Institute of Biochemistry and Cell Biology, Shanghai Institutes for Biological Sciences, Chinese Academy of Sciences, Shanghai, 200031 China; 4 Department of Pathology, Zhongshan Hospital, Fudan University, Shanghai, 200032 China

**Keywords:** Cell growth, Cancer epigenetics

Correction to: *Nature Communications* 10.1038/s41467-017-00350-9, published online 17 August 2017.

This article contained errors in Figs. 1, 4, Supplementary Fig. 3 and Supplementary Fig. 5. The correct data for Figs. 1 and 4 were included in the original full scan blots provided as Supplementary Information in the published article.

All western blot data from the Fig. 1a, 1b (shown below) were duplicated from Fig. 2b and Supplementary Fig. 1c, respectively.



The correct versions of Fig. 1a and 1b were shown in the original full scan blots in Supplementary Fig. 6. The correct version of Fig. 1 is shown below.



In the original Fig. 4d (shown below), the band showing β-actin expression in MDA-MB-231 cells was duplicated from Fig. 2b β-actin expression in MDA-MB-231 cells.

The correct data for this figure was included in the full scan of Supplementary Fig. 7a, which was probed for HEPH and GAPDH as a control and is shown below.

All mentioned changes to the original version were corrected in the pdf and html versions of the Article

In the original version of Supplementary Fig. 3b (shown below), the representative image of comet assay in group MCF-7 UNC 5 µm +FAC 100 µm was duplicated from the image of MCF-7 Con.

The correct data for this figure is now shown below.

In the original version of Supplementary Fig. 5, the G9a band in MDA-MB-231 cells was duplicated from the G9a band in MDA-MB-231 cells in Fig. 1a. Due to damage to the hard drive storing the original data, we are unable to provide the source data. We repeated the experiments and the data for Supplementary Fig. 5e is shown in the Supplementary Information file with this correction. In this experiment β-actin is provided as the loading control instead of GAPDH.

In addition, we also provide contemporaneous independent repeats of the experiments shown in Fig. 2b in the Supplementary Information file with this correction.

## Supplementary information

Supplementary Information

