# Supplementary material for: G9a regulates breast cancer growth by modulating iron homeostasis through the repression of ferroxidase hephaestin
Source: Nat Commun. 2017 Aug 17;8:274. doi: 10.1038/s41467-017-00350-9 (PMC5561105; doi:10.1038/s41467-017-00350-9)
Supplement: Supplementary file 1 — Supplementary Information [file 41467_2017_350_MOESM1_ESM.pdf]

Description of Supplementary Files

File Name: Supplementary Information
Description: Supplementary Figures and Supplementary Tables

File Name: Peer Review File

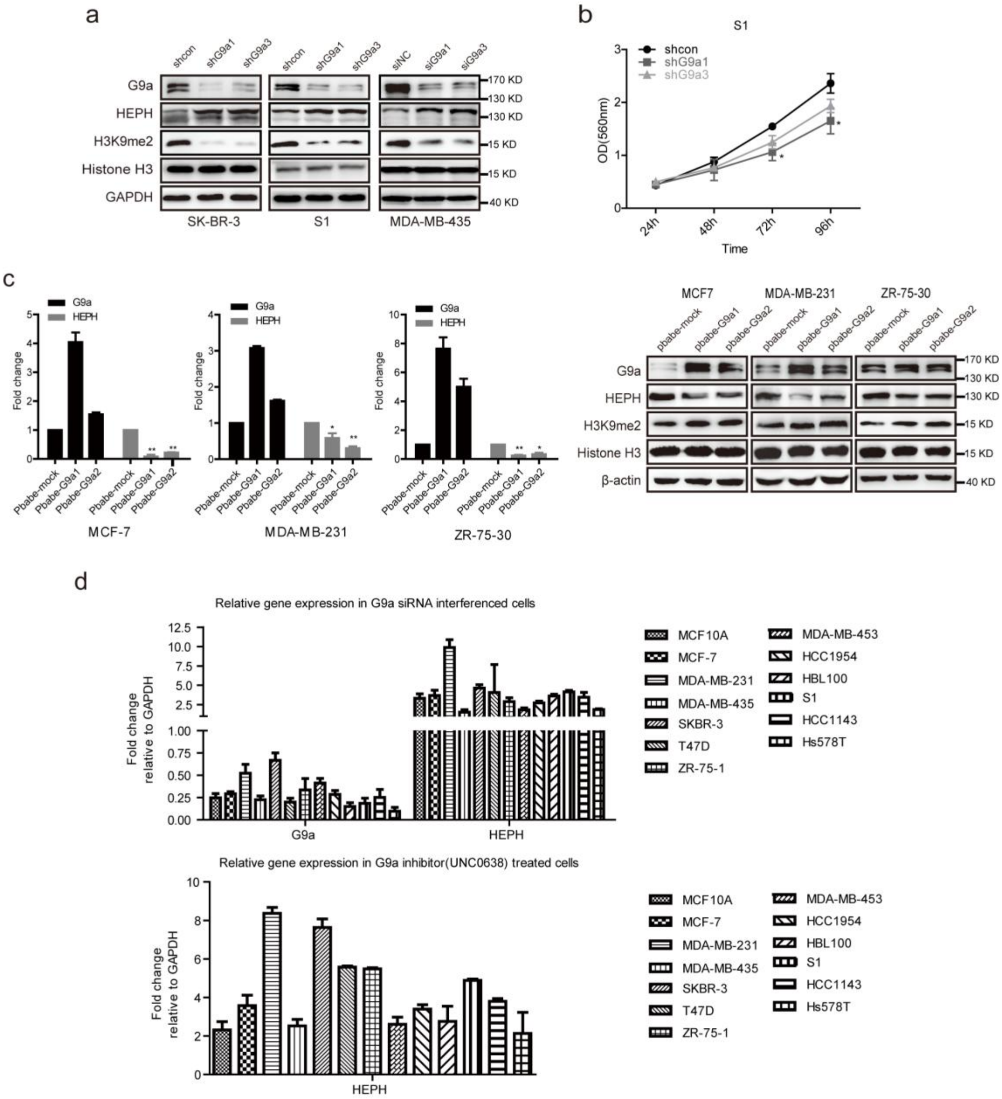

Supplementary Figure 1. G9a repressed HEPH expression.

(a) G9a depletion with HEPH upregulation in SK-BR-3, S1 and MDA-MB-435 cells was analyzed by western blotting. **(b)** Depletion of G9a in S1 cells delayed cell proliferation *in vitro*. **(c)** Relative HEPH mRNA and protein levels in G9a over-expressed cells were analyzed by RT–PCR and western blot **(d)** G9a knockdown or inhibition results were confirmed with ten more cell lines, and HEPH mRNA was increased in all these G9a depletion cell lines. The results presented are from three independent experiments. Error bars indicated ± s.d. (*P < 0.05, **P < 0.01, ***P < 0.001, Student's t-test paired).

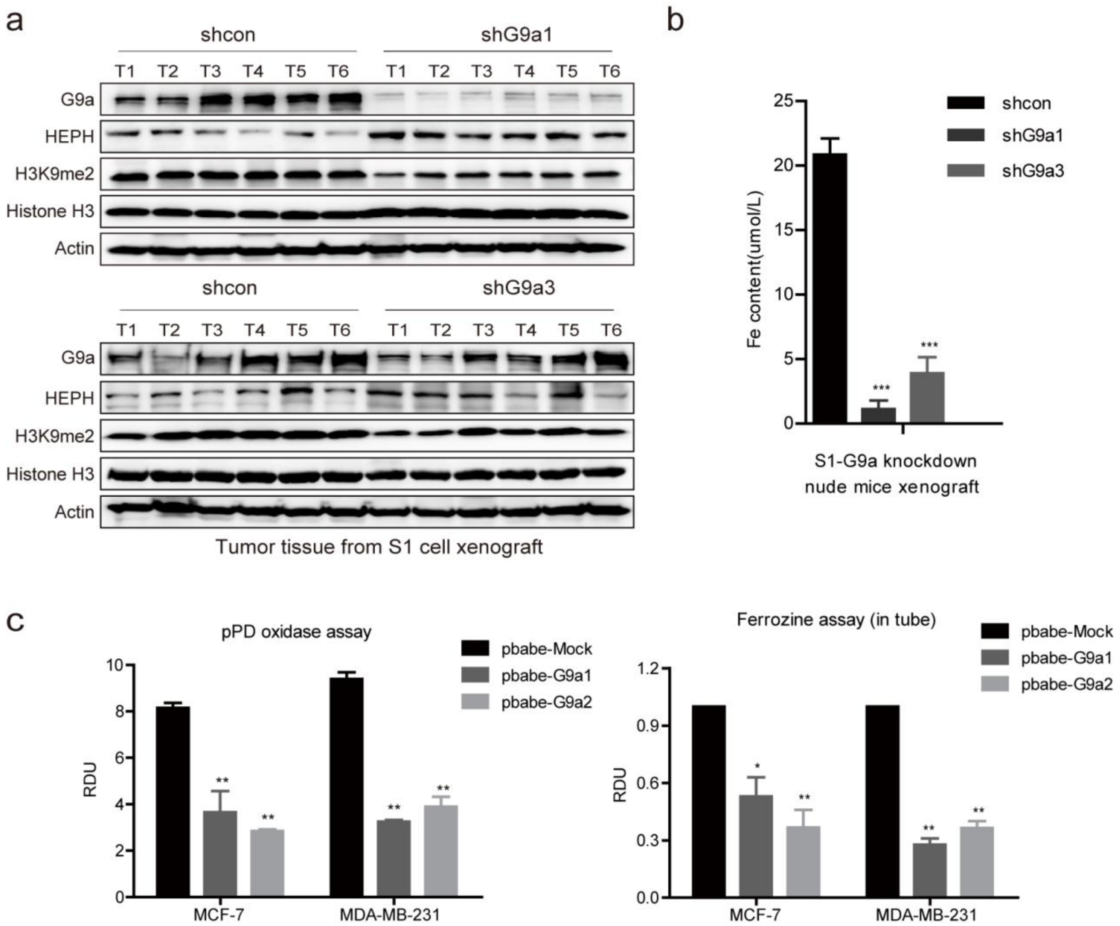

Supplementary Figure 2. . G9a-regulated HEPH expression led to a decrease of iron in vivo.

(a) Western blotting analysis showed that loss of G9a increased HEPH protein levels in S1 xenograft tumor tissues. **(b)** The Fe content in the xenograft tissues was measured using the clathrate color-display method. A significant decrease of non-heme iron content was found in the G9a-depleted S1 tumor tissue. **(c)** HEPH activity was measured by the oxidation of pPD and ferrozine assays in MCF-7 and MDA-MB-231 G9a-overexpressed cells. The results presented are from three independent experiments. Error bars indicated ± s.d. (*P < 0.05, **P < 0.01, ***P < 0.001, Student's t-test paired).

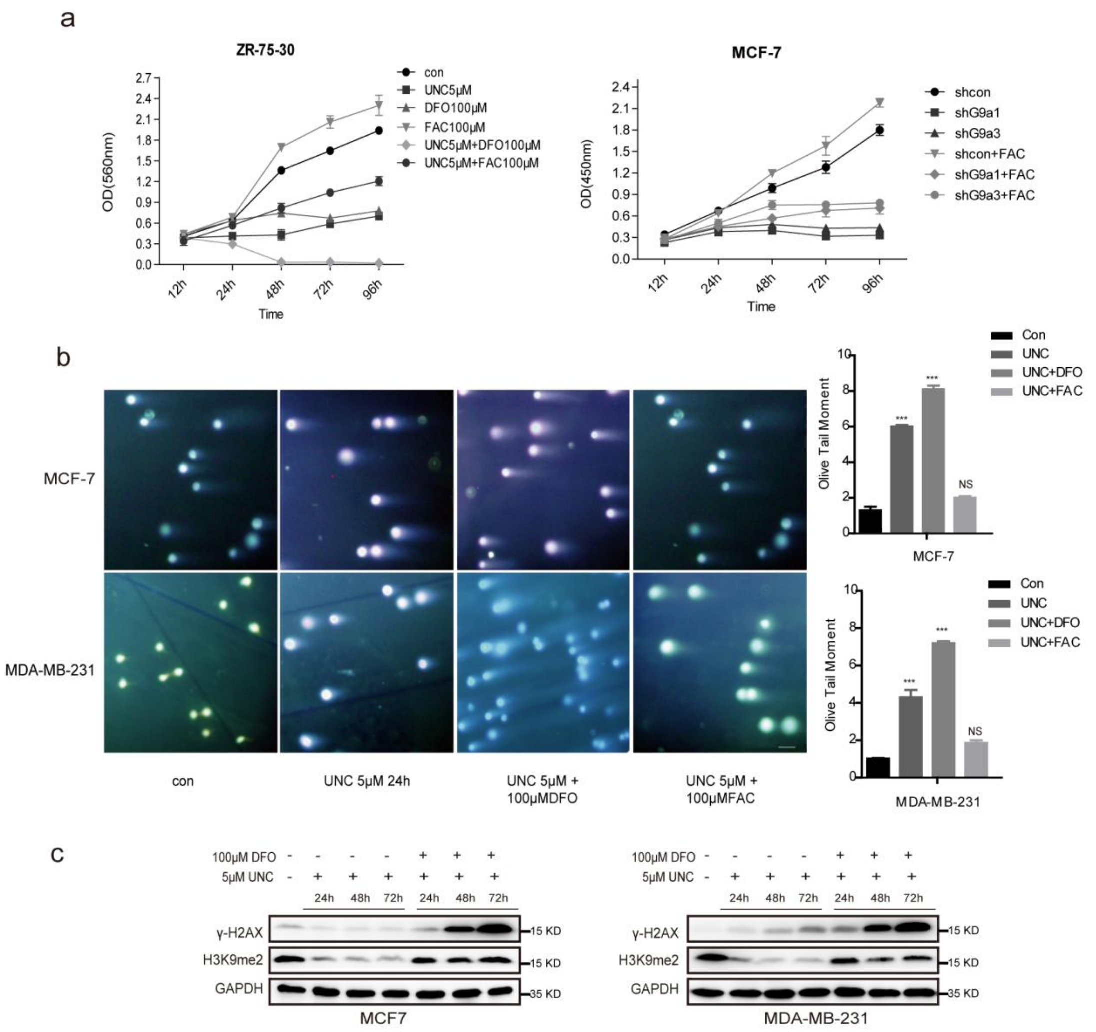

Supplementary Figure 3. DNA damage caused by G9a inhibition was enhanced or reduced under iron depletion or overload.

(a) Growth curves of ZR-75-30 and MCF-7 cells with or without G9a inhibitor treatment and G9a knockdown cell lines cultured in standard media or media supplemented with 100 μM desferrioxamine (DFO) or 100 μM ferric ammonium citrate (FAC). DNA damage as assessed by the comet assay and tail moment quantification **(b)** (scale bar 25 μm) or western blotting analysis of the γH2AX protein level **(c)**. The results presented are from three independent experiments. Error bars indicated ± s.d. (*P < 0.05, **P < 0.01, ***P < 0.001, Student's t-test paired).

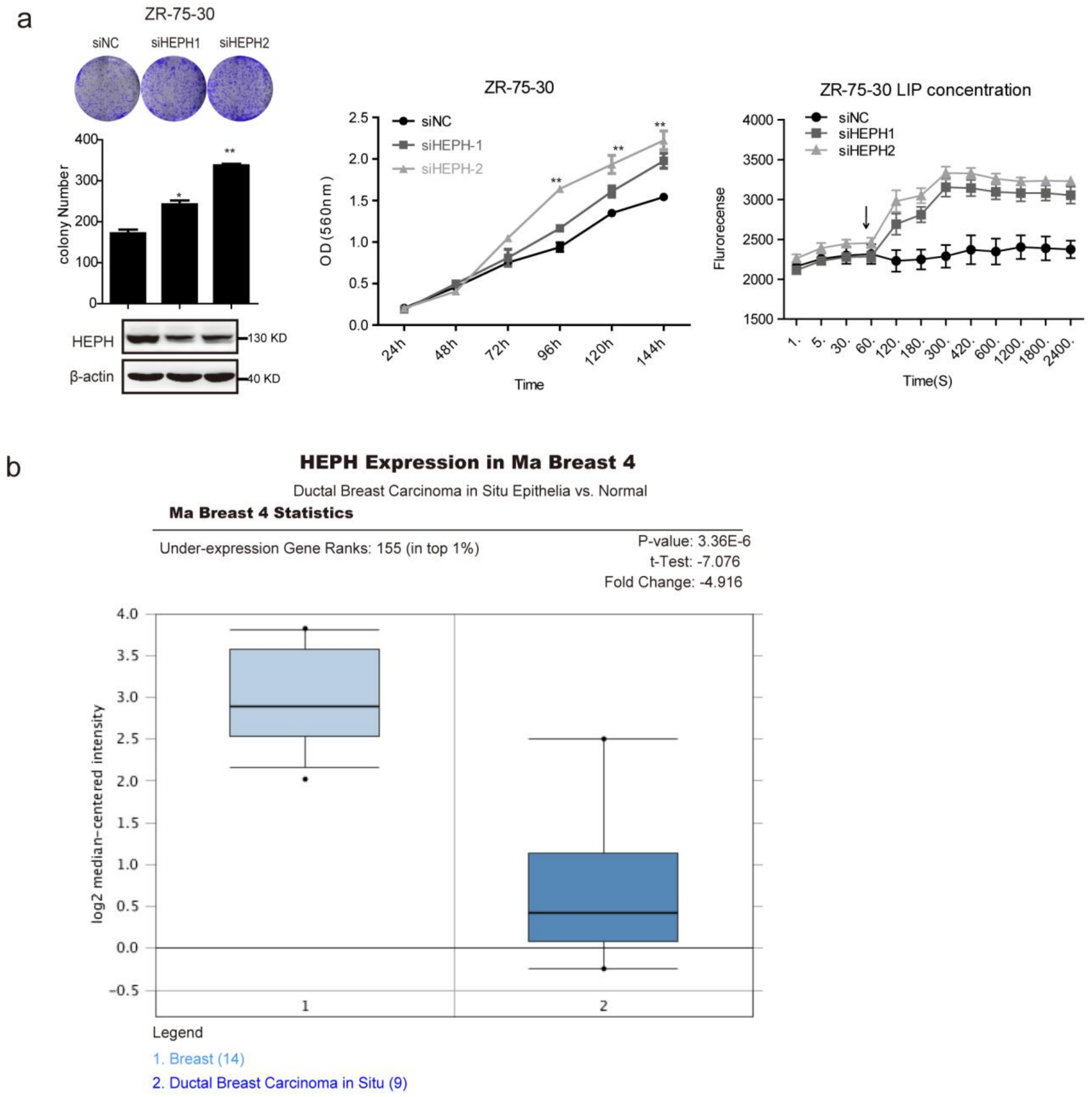

Supplementary Figure 4. Down-regulation of HEPH increased cellular iron content and accelerated breast cancer cell proliferation.

(a) Clonogenic assays, cell growth and labile iron pool concentration of ZR-75-30 cells expressing control siRNA (siNC) or siRNA-targeted HEPH (siHEPH1 and siHEPH2) were analyzed. **(b)** HEPH expression in normal breast tissue and ductal breast carcinoma in Ma breast dataset from Oncomine database. The values of log2 median-centered intensity detected by indicated probe sets were displayed as a boxplot according to Oncomine output. Error bars indicated ± s.d. (*P < 0.05, **P < 0.01, ***P < 0.001, Student's t-test paired).

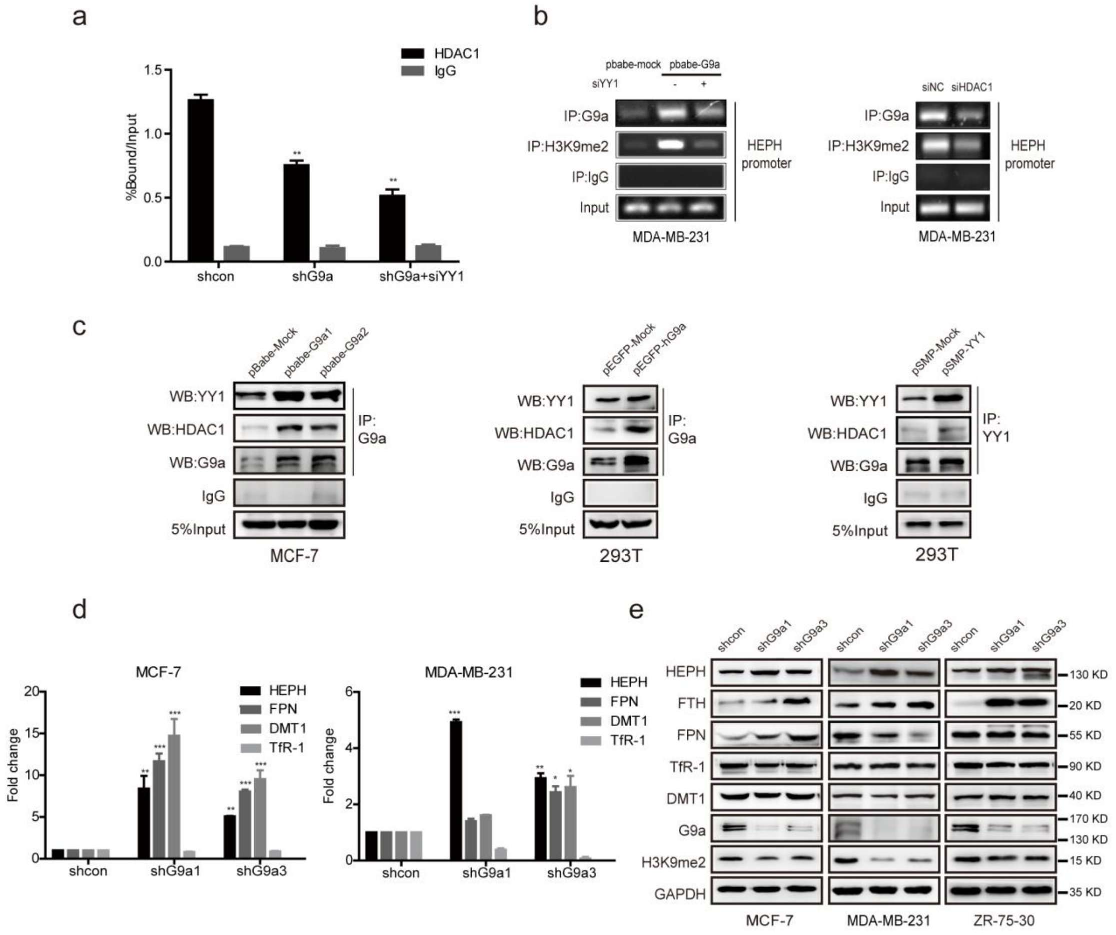

Supplementary Figure 5. G9a shows relatively little influence over other proteins relative to iron metabolic pathway.

(a) The abundance of HDAC in the *HEPH* Pro2 region was determined by ChIP in G9a knockdown cells or siYY1-treated cells. **(b)** RT-PCR was used to test the binding levels of G9a and HDAC in the Pro2 region of the *HEPH* promoter in G9a-overexpressed cells treated with siYY1 and siRNA-mediated HDAC1 knockdown cells. **(c)** G9a endogenously interacts with YY1 and HDAC1 in MCF-7 cells. Anti-G9a immunoprecipitates from MCF-7 whole-cell extracts were analyzed by western blotting analysis with anti-YY1 and anti-HDAC1 antibodies. HEK293T cells were transfected with pEGFP-G9a or pSMP-YY1, and anti-G9a or anti-YY1 immunoprecipitates were analyzed by western blotting analysis with anti-HDAC1 antibody. The mRNA **(d)** and Protein **(e)** levels of DMT1, FPN, and TfR1 from MCF-7, MDA-MB-231, and ZR-75-30 G9a knockdown cell lines were determined. Error bars indicated ± s.d. (*P < 0.05, **P < 0.01, ***P < 0.001, Student's t-test paired).

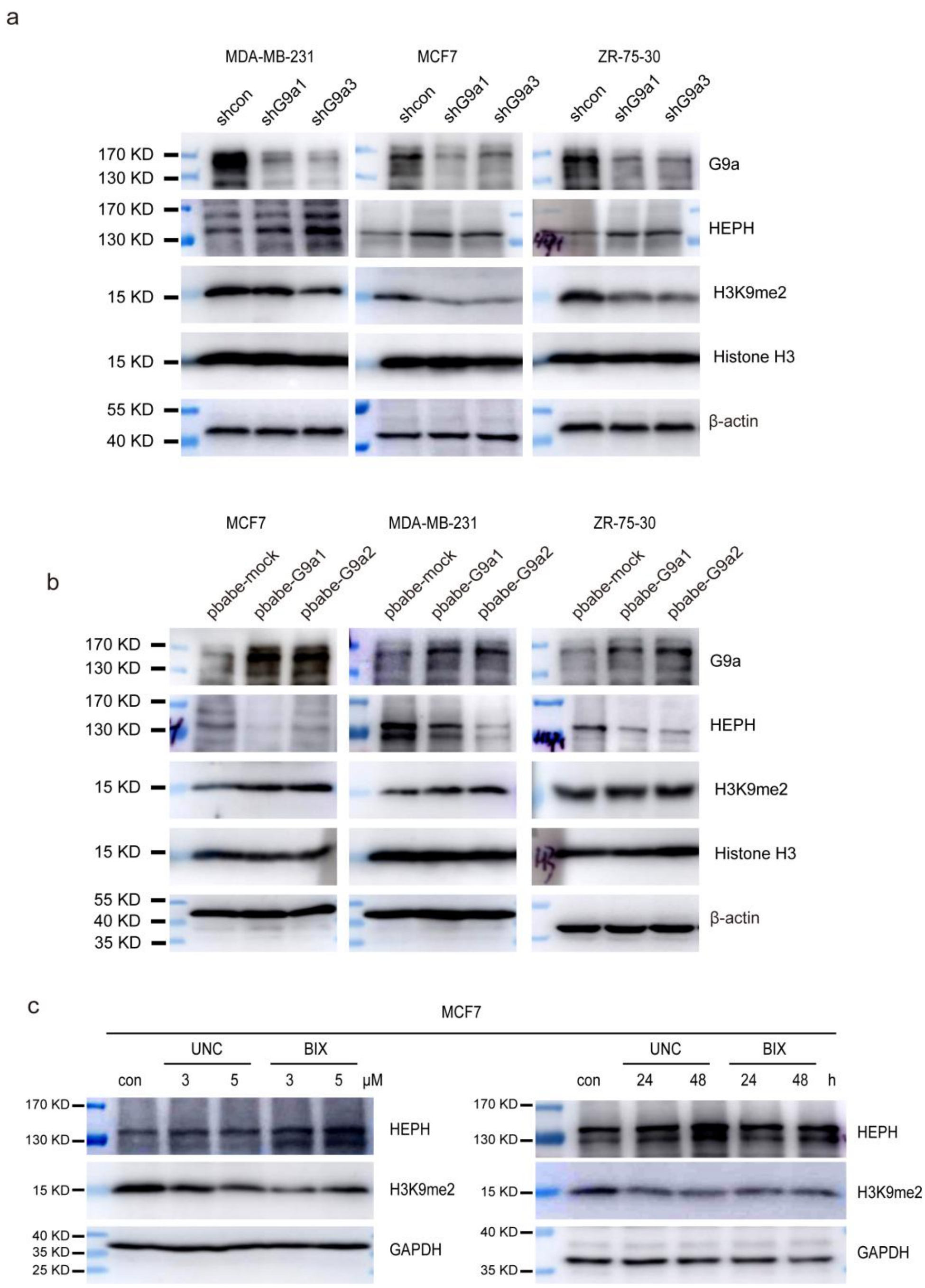

Supplementary Figure 6. Uncropped blot images of western blots analyses in Figure 1, Figure 2 and Supplementary Figure 1. (a) Figure 1a, Figure 2b. **(b)** Figure 1b, Supplementary Figure 1c. (c) Figure 2c.

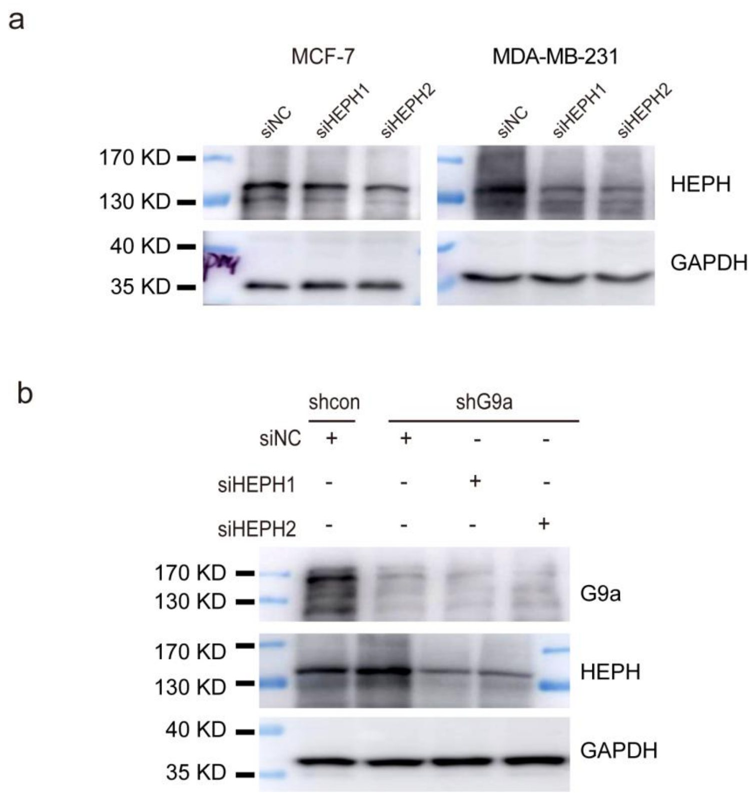

Supplementary Figure 7. Uncropped blot images of western blots analyses in Figure 4. (a) Figure 4d. **(b)** Figure 4g.

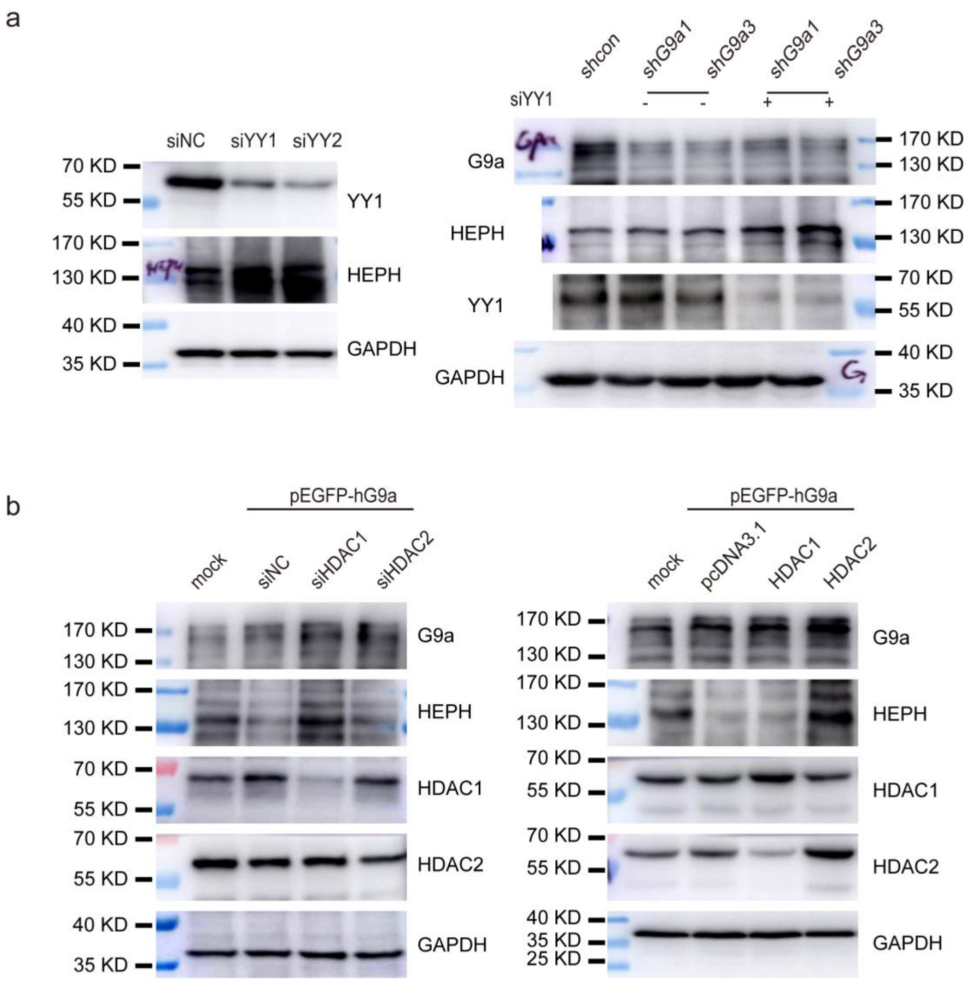

Supplementary Figure 8. Uncropped blot images of western blots analyses in Figure 6. (a) Figure 6a. **(b)** Figure 6e.

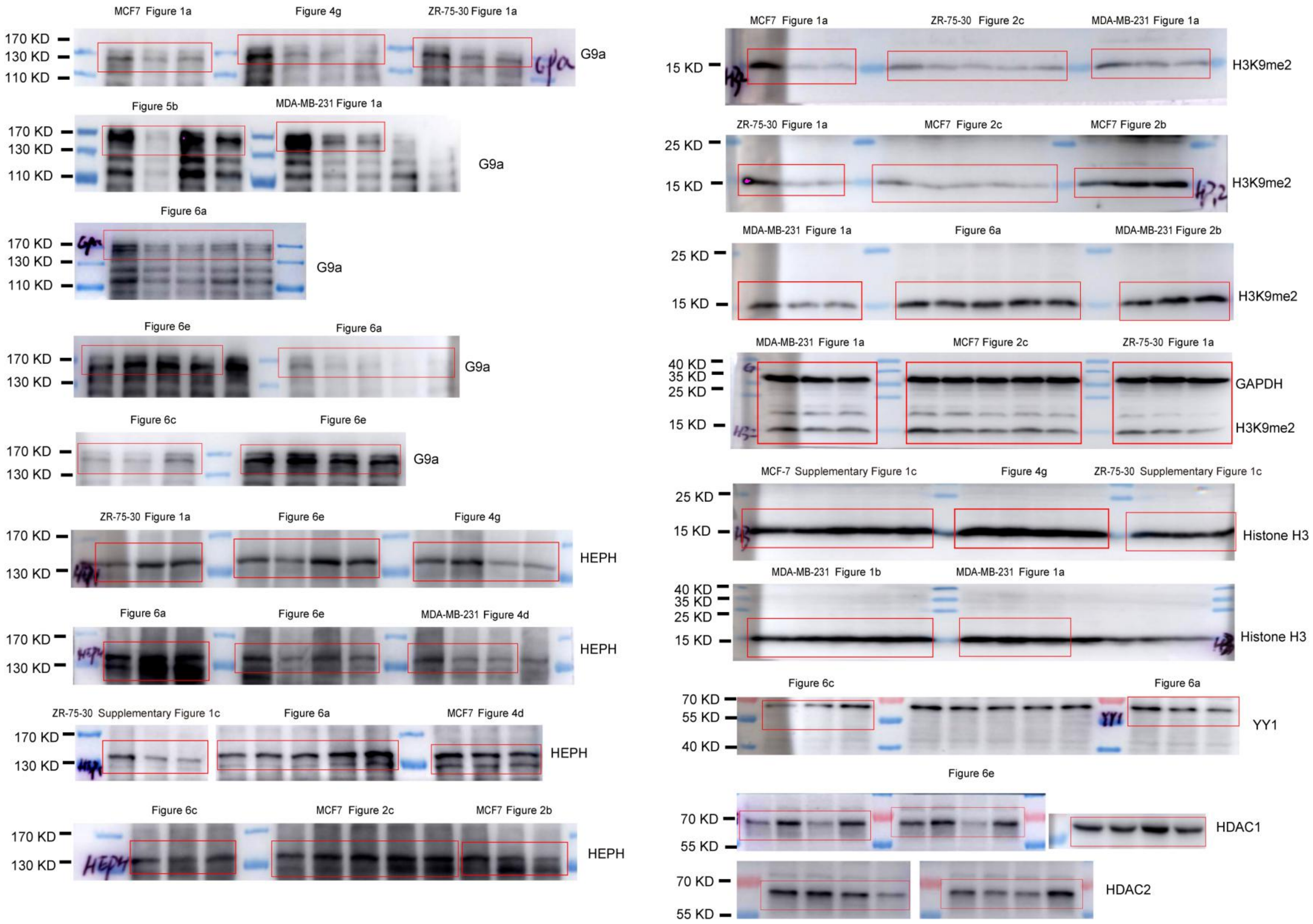

Supplementary Figure 9. Uncropped blot images of western blots analyses in the main figures.

Supplementary Table 1: Primers for cDNA fragment amplification (RT-PCR) assay

Gene name	Sequence (sense/antisense)
ferroportin	5'-ACCTCGCTGGTGGTACAGAATGTT-3'
	5'-AGCAGGAAGTGAGAACCCATCCAT-3'
hepcidin	5'-CCTGACCAGTGGCTCTGTTT-3'
	5'-CACATCCCACACTTTGATCG-3'
TfR-1	5'-ATCTCGGTCATCAGGATTGC-3'
	5'-CTGTGCCTACACCGGATTTT-3'
DMT1	5'-TGGCTTATCTGGGCTTTGTG-3'
	5'-CACACTGGCTCTGATGGCTA-3'

Supplementary Table 2: Primers for DNA fragment amplification (chromatin immunoprecipitation assay)

Primer name	Position	Length(bp)	Sequence (sense/antisense)
HEPH-Pro1	-2210—-1950	310	5'-CAGAAAGATTCTTGGGCAAAGG-3'
			5'-GTTTTTGCTTCAGTAAGTCTTGGG-3'
HEPH-Pro2	-1250—-870	330	5'-GATCCTGAGGTAAAGGGGTTAGACT-3'
			5'-TCAGCATGTTCAGACCCCCAGT-3'
HEPH-Pro3	-450—-95	305	5'-TCAGAAAAAGGCAGCCAAGATACA-3'
			5'-TATGACTGGTTTCCTAGACAATGTG-3'
HEPH-Pro4	-20—+230	140	5'- ATTATCTCCATTTGAAAACTGAGGT-3'
			5'-TGAAAAACATTTTAAAGTTGGTGTC-3'

Supplementary Table 3: Primer sequences for genomic DNA amplification (reporter gene assay)

Primer name	Position	Length(bp)	Sequence
HEPH-P1 F	-918	1168	5'-CG**ACGCGT**CTTCCCTGCCTTACCTAG-3'
HEPH-P2 F	-366	616	5’-CG**ACGCGT**GCTCTTAACAGTGATGCCATAT-3’
HEPH-P3 F	-113	363	5’-CG**ACGCGT**CTCCAGGAGAAGGAACCA-3’
HEPH-P R	+250		5'-CCG**CTCGAG**TGCTCAGTGGTCAAGGGA-3'
