# Supplementary material for: Author Correction: G9a regulates breast cancer growth by modulating iron homeostasis through the repression of ferroxidase hephaestin
Source: Nat Commun. 2020 Jul 24;11:3789. doi: 10.1038/s41467-020-17413-z (PMC7381662; doi:10.1038/s41467-020-17413-z)

Supplementary Information

Repetitive data 1:

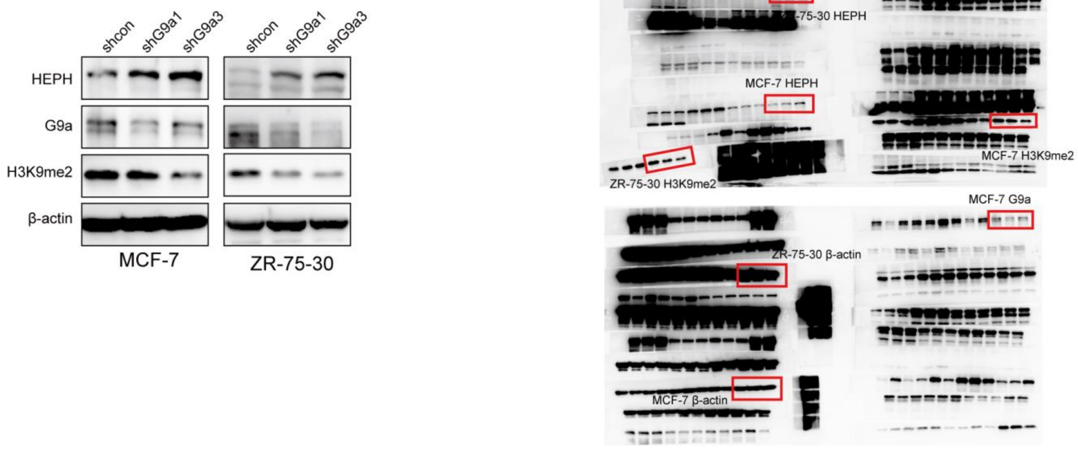

Repetitive data 2:

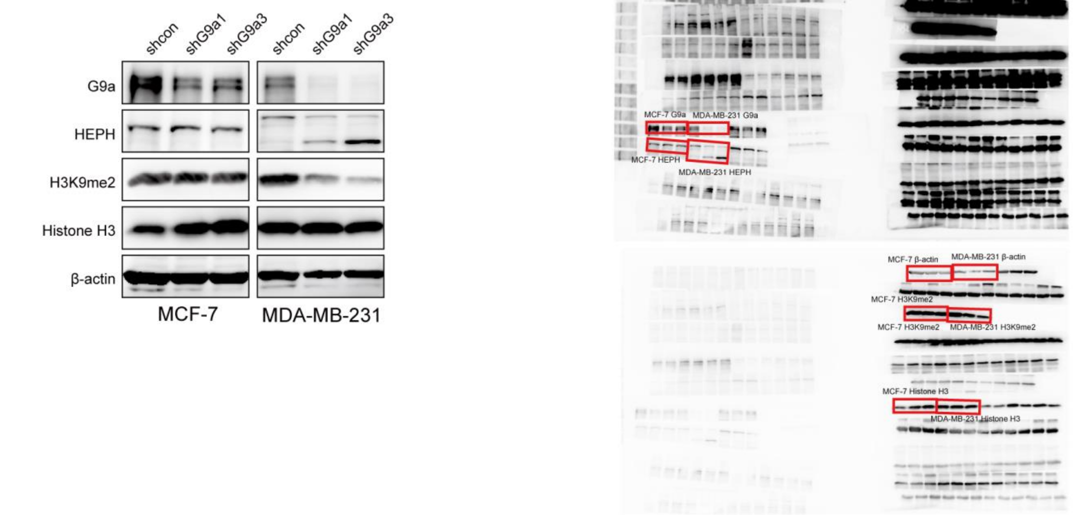

Repetitive data 3:

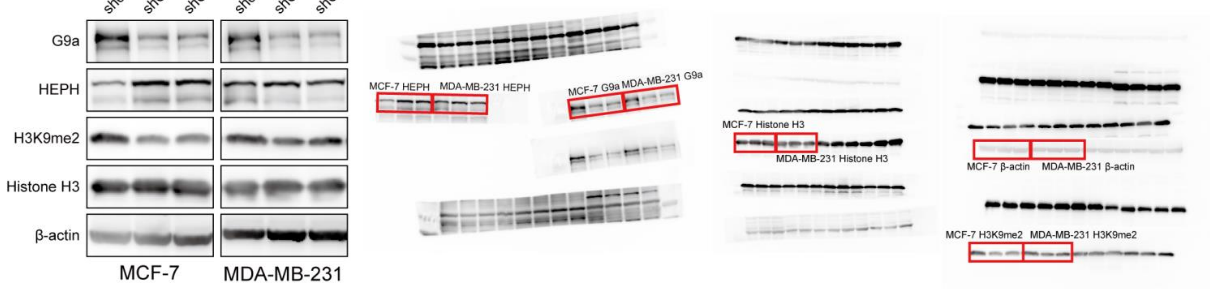

Figure S5e full uncropped blots:

File name:20191209_1048

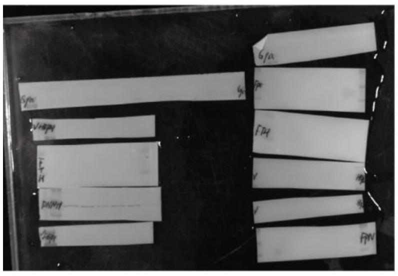

File name:20191209_1048_1

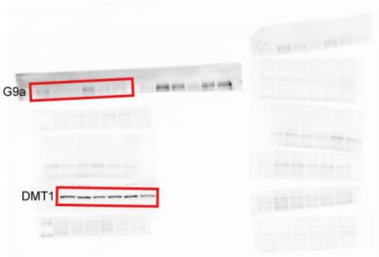

File name:20191209_1048_7

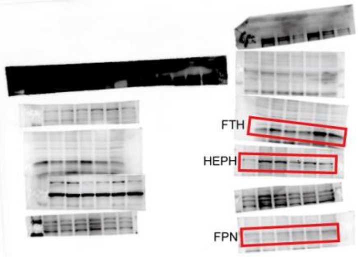

File name:20191209_1049

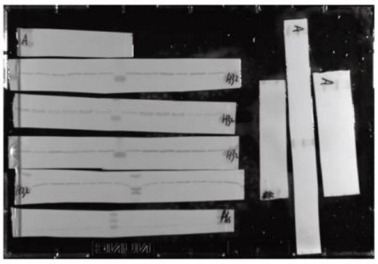

File name:20191209_1053_1

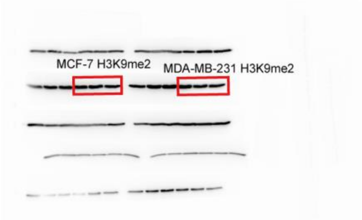

File name:20191209_1053_8

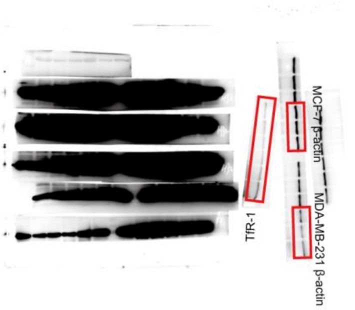

File name:20191209_1017

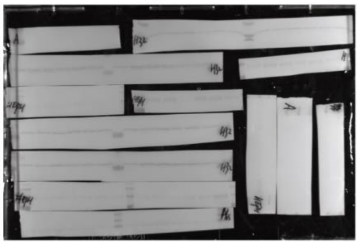

File name:20191209_1015

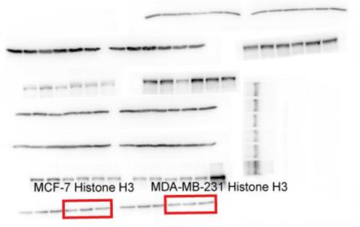

Supplement: Supplementary file 1 — Supplementary Information [file 41467_2020_17413_MOESM1_ESM.pdf]
